# Supplementary material for: A set of multi-entry identification keys to African frugivorous flies (Diptera, Tephritidae)
Source: Zookeys. 2014 Jul 24;(428):97–108. doi: 10.3897/zookeys.428.7366 (PMC4143993; doi:10.3897/zookeys.428.7366)
Supplement: Supplementary material 6 — Key to Ceratitis [file zookeys-428-097-s006.zip › SF6_ZooKeys_key to Ceratitis/key/SF6_ZooKeys_key to Ceratitis/Media/Html/Ceratitis copelandi.htm]

Ceratitis (Pterandrus) copelandi, n


***Ceratitis*** **(*Pterandrus*) *copelandi* De Meyer & Freidberg**

Body
length: 4.40 (4.20-4.60) mm; wing length: 4.90 (4.45-5.50) mm.

Male

Head: Antenna yellow or orange (first flagellomere partly
brown in Zimbabwe specimen). First flagellomere three times as long as pedicel.
Arista with short rays; ventral rays slightly shorter and sparser than dorsal
rays, especially basally. Frons convex with longitudinal depression, occellar
triangle at slight elevation but not distinct protuberance as in tananarivana;
frons orange, with median silvery vitta from ocellar triangle along depression
to anterior margin, especially visible well when viewed from anteroventral
angle; with short scattered setulae largely of same color as frons. Frontal
setae intermediate in size between anterior and posterior orbital setae. Face
yellowish white. Genal seta and setulae dark, well developed.

Thorax: Postpronotal
lobe white, unspotted. Mesonotum dark ground color, blackish; with streaks and
darker markings but without distinct spots or clearly defined stripes except
yellowish prescutellar markings, which are separate but with pale area in
between; microtrichial pattern silvery gray; pilosity pale. Scapular setae
black. Scutellum yellowish white, basally without spots, at most slightly
darker coloration, occasionally more pronounced; apically with three separate
black spots, covering apical 0.6. Anepisternum on ventral half brownish yellow;
with pale pilosity (not as dense and silvery as in *tananarivana*).

Legs:
Yellow (in Zimbabwe specimen more yellowish orange) except where otherwise
noted; setation typical for subgenus, mixed pale and dark. Foreleg: femur
darkened; posteriorly with black and pale setulae; ventral setae long and
black; tibia slightly flattened dorsoventrally and expanded laterally, white
(in Zimbabwe specimen darker), with short white setulae. Midleg: tibia partly
flattened dorsoventrally, distal 0.4 broader; tibia mostly yellow but broadened
part blackish, weakly silvery when viewed from certain angle, anteriorly with
pale setulae; with black feathering dorsally and ventrally on distal 0.4; tarsi
normal. Hindleg: femur at apical 0.25 with longer pale setae dorsally,
and dark setae ventrally.

Wing:
bands yellowish brown to brown. Interruption between marginal and discal bands
around end of vein R_1_ clear and complete; cubital band free; medial
band free but narrower than in figure 14, in some Kenyan specimens poorly
developed; crossvein R-M just proximal to middle of discal cell. Apex of vein R_1_
distal to level of crossvein R-M. Crossvein DM-Cu slightly oblique
anterodistally.

Abdomen:
Tergites mainly dark brown, tergite 5 sometimes partly yellowish brown.
Tergites 2 and 4 silvery across posterior half. Male epandrium in lateral view
moderately broadened; with posterior lobe of lateral surstylus short and
slightly curved.

Female

As
male except for the following characters: Ocellar triangle not on elevation.
Frontal longitudinal depression weak, but with distinct silvery vitta present.
Legs without feathering; pilosity of forefemur mixed white and dark. Oviscape
shorter than preabdomen. Aculeus at most six times longer than wide; tip with
distinct apical indentation and lateral margin slightly sinuous.

(Description
after De Meyer & Freidberg, 2006)
